# Multi-Omics Uncover the Mechanism of Wheat under Heavy Metal Stress

**DOI:** 10.3390/ijms232415968

**Published:** 2022-12-15

**Authors:** Min Zhou, Shigang Zheng

**Affiliations:** 1 School of Life Sciences, Chongqing University, Chongqing 401331, China; 2 Center of Plant Functional Genomics, Institute of Advanced Interdisciplinary Studies, Chongqing University, Chongqing 401331, China; 3 Chengdu Institute of Biology, Chinese Academy of Sciences, Chengdu 610041, China

**Keywords:** wheat, heavy metal stress, omics, multi-omics, functional genes

## Abstract

Environmental pollution of heavy metals has received growing attention in recent years. Heavy metals such as cadmium, lead and mercury can cause physiological and morphological disturbances which adversely affect the growth and quality of crops. Wheat (*Triticum aestivum* L.) can accumulate high contents of heavy metals in its edible parts. Understanding wheat response to heavy metal stress and its management in decreasing heavy metal uptake and accumulation may help to improve its growth and grain quality. Very recently, emerging advances in heavy metal toxicity and phytoremediation methods to reduce heavy metal pollution have been made in wheat. Especially, the molecular mechanisms of wheat under heavy metal stress are increasingly being recognized. In this review, we focus on the recently described epigenomics, transcriptomics, proteomics, metabolomics, ionomics and multi-omics combination, as well as functional genes uncovering heavy metal stress in wheat. The findings in this review provide some insights into challenges and future recommendations for wheat under heavy metal stress.

## 1. Introduction

Heavy-metal-driven contamination of agricultural soil is serious, causing worldwide environmental and public health issues [1,2]. Metal-contaminated agricultural soil may comprise two classes of heavy metals. Toxic elements such as cadmium (Cd), lead (Pb) and mercury (Hg) are highly toxic to living cells even at very low concentrations [3,4]. Another class, essential micronutrients such as zinc (Zn), iron (Fe), copper (Cu), nickel (Ni) and manganese (Mn) play key roles in several metabolic reactions in plants and, hence, can exhibit beneficial effects on plant growth at an optimum concentration [5,6,7,8]. However, they prevent other nutrients from performing normal metabolic functions, leading to detrimental effects in plants when present in excess quantities [9,10,11,12,13,14]. For example, it has been reported that Cu participated in lignin biosynthesis in plants at the optimum amount, while leading to lipid peroxidation through increasing malondialdehyde contents at an excessive amount [14]. Heavy metal contaminations generally occur from human activities including the excessive use of pesticides and fertilizers, mining, the manufacturing industry and sewage discharge [15]. Heavy metals in soils or waters can be transferred through the food chain, then can be accumulated in plants and animals and eventually transferred to the human system, posing a threat to human health [16,17,18]. Compared to other heavy metals, Cd, Hg, Pb and Cu are regarded as environmental hazards because they are toxic for plants and humans as well as other living organisms [19,20,21].

In recent years, the issue of excessive accumulation of heavy metals in agricultural products has attracted more and more attention due to heavy metal contaminations of agricultural soils and the detrimental effects they have on living organisms [22]. For humans, excessive heavy metal intake damages the kidneys, liver, heart, thyroid and bones, and their accumulation in human organs causes various diseases such as emphysema, renal and hepatic dysfunction, osteoporosis and cardiovascular disease [23,24,25,26,27]. For example, Pb and Cd may increase the liver’s metabolic burden, and lead to unusual liver function according to the association between blood Pb/Cd level and elevated hematological and hepatic parameters in patients from the exposed and control groups [24]. For plants, heavy metal toxicity can result in physiological, morphological and structural disturbances, and eventually adversely affect growth [28,29,30,31,32]. For example, it has been reported that Cd stress severely affected wheat root growth and morphology [33,34]. Wheat is one of the most important crops, occupying 22% of cultivated land [35] and acting as the primary food source for 60% of the world’s population [36]. Wheat plants have revealed a high susceptibility to accumulating high contents of heavy metals (e.g., Cd, Pb and Cu) [37,38,39]. The Codex Alimentarius (CDX 1993–1995, Amended 2019) has established a maximum level of 0.2 mg kg^−1^ for both Cd and Pb in wheat [40]. Wheat plants experience various environmental stresses across their lifecycle and have, consequently, had the capacity to adapt to these stresses [41]. Among these stresses, heavy metals have received growing attention in recent decades. To deal with heavy metal stresses such as Cd, Pb, Fe, Hg and Zn stresses, wheat plants have induced either avoidance of uptake or tolerance mechanisms including enhancement of the activities of antioxidant enzymes, regulation of ion homeostasis, activation of certain genes and production of stress proteins [42,43,44,45,46]. In addition, several strategies, such as exogenous use of plant growth regulators and nanoparticles, application of inorganic amendments, organic amendments and biological entities, have been used for the management of heavy metal toxicity in wheat at the experimental stage [36,42,47,48]. Emerging advances in our understanding of factors that affect heavy metal uptake and accumulation, toxicity and tolerance mechanisms against heavy metal in wheat have been made very recently [36]. Especially, the molecular mechanisms of wheat under heavy metal stress are increasingly being recognized. The findings in this review provide some insight into the mechanism of wheat under heavy metal stress through summarizing the recently described epigenomics, transcriptomics, proteomics, metabolomics, ionomics and multi-omics combination, as well as functional genes in wheat under heavy metal stress.

## 2. Epigenomics and Heavy Metal Stress in Wheat

**Figure 1:**
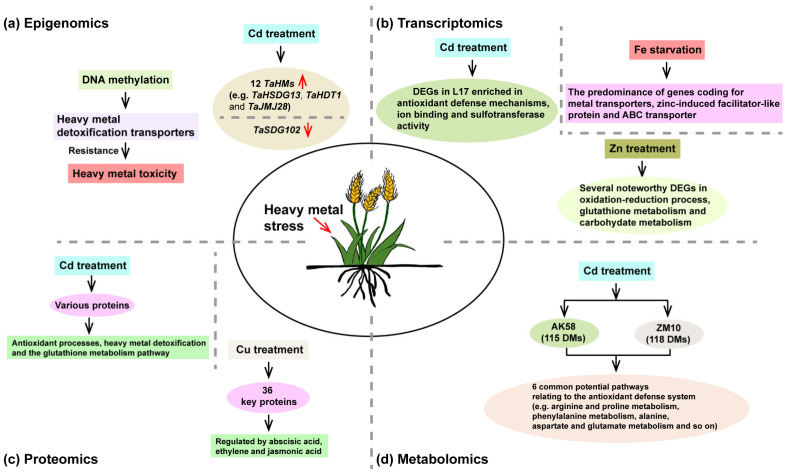
Multi-omics reveal the mechanism of wheat under heavy metal stress. Epigenomics (**a**), transcriptomics (**b**), proteomics (**c**) and metabolomics (**d**) reveal heavy metal stress in wheat. DEGs, differentially expressed genes. DMs, differential metabolites. Up red arrow means upregulated, down red arrow means downregulated.

Epigenetics is currently defined as the study of changes in gene expression that are heritable and induced by methylation of DNA, post-translational modifications of histones than an alteration in the underlying sequence of DNA [49]. Epigenomics are the mechanisms that play an important role in the regulation of the genome through several processes of epigenetics [50]. A growing body of evidence points to the importance of epigenetic modifications in response to heavy metal stress in plants. Heavy metal stress, such as Cd stress, Pb stress and Zn stress, can induce dramatic changes in the epigenome of plants [51,52,53]. However, the study of the epigenome in response to heavy metal stress in wheat is limited. For instance, it has been reported that CG DNA hypomethylation was at the promoter region of *TaHMA2* (Heavy Metal ATPase 2, HMA2) and *TaABCC2/3/4* (ATP-Binding Cassette) metal detoxification transporters in Pb-, Cd- and Zn-resistant wheat (Pirsabak, 2004) under Pb, Cd and Zn treatments compared with a Pb-, Cd- and Zn-sensitive variety (Fakhar-e-sarhad) and non-treatment control [41]. *TaHMA2* plays an important role in the long-distance transport of Cd and Zn in wheat [54]. Their result suggested that DNA methylation modulated the expression of heavy metal detoxification transporters to show resistance against heavy metal toxicity [41] (Figure 1a). For histone modification, Cd treatment led to a 2.2- to 6.4-fold increase in the expression levels of 12 histone modification genes (*TaHMs*) such as *TaSDG13*, *TaHDT1* and *TaJMJ28* in the roots of wheat, but reduced the expression of *TaSDG102* [55] (Figure 1a). Moreover, it has recently become clear that some traits can be inherited by the modification of DNA (such as DNA methylation) that do not change its sequence [50]. Epigenetic quantitative trait loci (epiQTL) mapping can effectively find the epigenetic effects of loci related to phenotypic variations for target traits [56], and epiQTLs can be applied in epibreeding [57]. EpiQTLs have been identified in *Arabidopsis thaliana* and *Populus* [56,58]. However, the study about epiQTLs in wheat under heavy metal stress is unclear. Furthermore, epigenome editing uses progressive tools such as CRISPR-Cas9, which has participated in the targeted editing of the epigenome. This strategy has been studied in the model plant *Arabidopsis*, which has been edited for improved tolerance to drought stress [59], and to yield the late flowering phenotype and the developmental phenotype [60,61]. However, epigenome editing for the mitigation of heavy metal stress in wheat is still understudied.

In conclusion, substantial efforts are still required to investigate other epigenetic responses to heavy metal stress in wheat, such as RNA m^6^A modification and chromatin remodeling. It is also necessary to explore the epigenetic response to other heavy metal stress, such as Cu, Zn and Fe stress, in wheat, investigate epiQTLs in wheat under heavy metal stress, as well as editing the epigenome of wheat by CRISPR-Cas9 for improved heavy metal stress tolerance.

## 3. Transcriptomics and Heavy Metal Stress in Wheat

Transcriptomics is applied to investigate all types of RNA transcripts, including mRNAs, microRNAs and long noncoding RNAs, in any organism [62,63]. Several transcriptomic strategies are commonly applied to evaluate the importance of transcripts under heavy metal stress in plant species [64,65,66,67,68]. Heavy metal stress, such as Cd stress, Fe stress and Zn stress, can lead to dramatic changes in the transcriptome of wheat compared to the control group, with most insights garnered by RNA sequencing (RNA-seq) analyses [69,70,71,72,73,74,75,76]. It was shown that genes were differentially expressed and showed distinct expression patterns in the roots of the low-Cd-accumulating wheat genotype (L17) and high-Cd-accumulating wheat genotype (H17) after Cd treatment through RNA-seq analysis [69]. These results also indicated that differentially expressed genes related to antioxidant defense mechanisms, ion binding and sulfotransferase activity were more accumulated in L17 [69] (Figure 1b). Similarly, through RNA-seq analysis, another study also investigated the transcriptional profiling of wheat under Cd stress. These results demonstrated that genes involved in the homeostasis reactive oxygen species play a key role in wheat under Cd stress [70]. Moreover, a total of 5654 genes have been identified under Fe starvation conditions in wheat roots by RNA-seq analysis [73]. Among them, the predominance of genes coding for metal transporters, zinc-induced facilitator-like proteins and ABC transporters was noted [73] (Figure 1b). These results also indicated that glutathione was involved in wheat under Fe stress at both the transcriptional and enzymatic activity levels [73]. Furthermore, another study showed that a total of 172 unigenes were expressed in the roots of wheat (*Triticum polonicum* L.) under Zn treatment compared with the control group [76]. Among them, several noteworthy differentially expressed genes were grouped into the oxidation–reduction process (three upregulated), glutathione metabolism (four upregulated) and carbohydrate metabolism (eight upregulated and three downregulated) [76] (Figure 1b). All these findings could help to elucidate the heavy metal tolerance in wheat at the transcriptional level and address breeding programs in the future.

MicroRNAs are a class of short 20–24-nucleotides, single-stranded and noncoding RNAs that inhibit gene expression by complementarity to messenger RNAs and play important regulatory roles in many biological processes in eukaryotes [77,78]. Their roles are well established; however, limited knowledge exists on their response to heavy metal stress in wheat plants [71,79,80,81]. For instance, it has been reported that five miRNAs and their targeted genes were differentially expressed in roots and leaves of wheat seedlings under Cd stress [80]. Among them, miR398 may participate in the tolerance to oxidative stress by modulating its target CSD to involve in Cd stress in wheat [80]. Another study showed that a total of 25 microRNAs were differentially expressed in L17 after Cd treatment, while 70 Cd-induced differentially expressed microRNAs were found in H17 [79]. Targeted genes of differentially expressed microRNAs enriched in the PI3K-Akt signaling pathway were uniquely accumulated in L17, and targeted genes of differentially expressed microRNAs related to carbohydrate digestion and the absorption pathway were especially accumulated in H17 [79]. Substantial efforts are still required to research microRNAs in response to other heavy metal stress, such as Zn, Fe and Cu stress, in wheat.

Above all, although great progress has been achieved in the transcriptome in response to heavy metal stress in wheat, substantial efforts are still required to figure out key functional genes that control heavy metal stress in wheat.

## 4. Proteomics Advances in Wheat under Heavy Metal Stress

Proteomics is defined as the qualitative and quantitative composition of expressed proteins, as well as how the expressed levels change under different conditions [82]. Importantly, emerging data show that the proteome and post-translational modifications of proteins play an important role in plants under heavy metal stress [23,39,83]. Proteomic analysis has been widely applied in wheat under biotic stresses [84,85,86,87,88]. However, the study of proteomics in wheat under heavy metal stress is limited. For instance, it has been reported that Cd stress induced various proteins in wheat. These proteins are mainly involved in antioxidant processes, heavy metal detoxification and the glutathione metabolism pathway [89,90,91] (Figure 1c). Another study indicated that differentially expressed proteins were identified in roots in the presence of strain TJ6 (a heavy metal-immobilizing bacterium, *Enterobacter bugandensis*) in wheat under Cd and Pb stress [92]. These differentially expressed proteins were mainly enriched in peroxidase activity, protein-DNA complexes and DNA packaging complexes [92]. In addition, Cu stress also induced dramatic changes in the proteome in the roots and leaves of wheat through the 2-DE method [21]. Most proteins were significantly increased in signal transduction, stress defense and energy production, while some proteins which participated in protein metabolism, carbohydrate metabolism and photosynthesis were severely decreased. These results also indicated that the Cu-responsive protein interaction network revealed 36 key proteins, most of which may be modulated by abscisic acid, ethylene and jasmonic acid [21] (Figure 1c). Substantial efforts are still required to investigate proteins in response to other heavy metal stresses, such as Zn and Fe stress, in wheat.

## 5. Metabolomics Uncovering Global Metabolic Changes in Wheat with Heavy Metal Stress

The metabolome contains all small molecules including primary and secondary metabolites in the cell or organism [62]. Metabolomics investigates global metabolic changes that can directly show the function of genes; thus, it can effectively exhibit chemical, biological and molecular mechanisms [93,94]. Metabolomics combined with other disciplines plays an important role in comprehending the interrelated biological processes linked to phenotypes [35]. Metabolomic analysis has been widely applied in wheat under abiotic stresses [95,96,97,98,99,100,101]. However, the study of metabolomics in wheat under heavy metal stress is limited. For example, it has been reported that the metabolomics profile in the roots of two wheat genotypes, AK58 (Aikang58, a grain low-Cd-accumulating genotype) and ZM10 (Zhenmai10, a grain high-Cd-accumulating genotype), under Cd stress [102]. These results showed that 115 and 118 differential metabolites were between control and Cd stress for AK58 and ZM10, respectively (Figure 1d). Through KEGG analysis, six common potential pathways relating to the antioxidant defense system, such as arginine and proline metabolism, phenylalanine metabolism, alanine, aspartate and glutamate metabolism, arginine biosynthesis, isoquinoline alkaloid biosynthesis, as well as glyoxylate and dicarboxylate metabolism, were identified in AK58 and ZM10 (Figure 1d). Moreover, another study reported that *Enterobacter bugandensis* TJ6 significantly decreased the Pb and Cd contents in the roots and leaves of wheat compared to the control group [92]. Through metabolomic analysis, a total of 248 metabolites were detected in the Pb/Cd-treated sample with strain TJ6 treatment. These results showed that these metabolic activities played a vital role in the tolerance of strain TJ6 to Pb and Cd [92]. Furthermore, another report showed that boron application reduced Cd concentrations in shoots and roots in wheat. Through metabolite analysis, they found that galactaric acid, citric acid, N6-galacturonyl-L-lysine and D-glucose were up-accumulated in boron application (boron + Cd) roots, while threoninyl-tryptophan and C16 sphinganine were down-accumulated [103]. In conclusion, more work is needed to figure out the metabolomic changes in wheat under other heavy metal stress, such as Zn, Fe and Cu stress.

## 6. Ionomics for Wheat under Heavy Metal Stress

Ionomics is a high-throughput elemental profiling method to study the ionome, where ionome is defined as “the mineral nutrient and trace element composition of cellular and living systems” [104,105]. For ionomic analysis, samples are typically digested in concentrated acid and diluted prior to analyzing. Then, inductively coupled plasma spectroscopy, either mass spectroscopy (ICP-MS) or optical emission spectroscopy (ICP-OES) is applied to simultaneously measure dozens of elements [106]. Ionomic analysis offers a powerful strategy for the functional analysis of the genes and gene networks that regulate the ionome and physiological processes that indirectly participate in controlling the ionome [104,105,106]. Ionomics also considers the changes in mineral composition in response to stress tolerance, such as salt stress, drought stress or cold stress [107,108,109]. Recently, for heavy metal stress in wheat, Hua et al. found through ICP-MS-assisted ionomics analysis that low-Fe stress significantly reduced the absorption of nutrients, including N, P, K, Mg, Ca, Fe, Mn, Cu, Zn and B nutrients in wheat [72]. Another study indicated that the Cd-sensitive wheat cultivar ZM32 had a higher root-to-shoot translocation coefficient of Cd and enriched more Cd in the grains than the Cd-resistant wheat cultivar ZM1860 through ionomic analysis [110].

Above all, the study of ionomics in wheat under heavy metal stress is not fully investigated, and the connection between cellular mineral nutrients and living systems in wheat under heavy metal stress is totally unclear.

**Figure 2:**
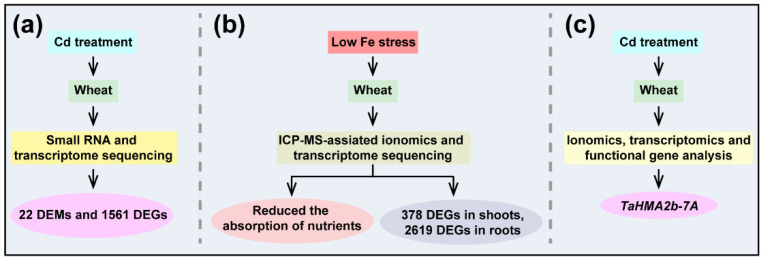
Combined multi-omics to study wheat under heavy metal stress. (**a**) In all, 22 differentially expressed microRNAs (DEMs) and 1561 differentially expressed genes (DEGs) were identified in a low-Cd-accumulating wheat cultivar through small RNA and transcriptome sequencing. (**b**) Low-Fe stress significantly reduced the absorption of nutrients through ICP-MS-assisted ionomic analysis; 378 and 2619 DEGs were found in the shoots and roots of wheat under low-Fe conditions. (**c**) *TaHMA2b-7A*, an important Cd exporter regulating long-distance Cd translocation, was identified in wheat through combining ionomics, transcriptomics and functional gene analysis.

In addition to single-omics, with the development of epigenomics, transcriptomics, proteomics, metabolomics and ionomics, researchers also have combined multi-omics to investigate the potential mechanism of wheat under heavy metal stress [71,72] (Figure 2). For instance, Zhou et al. reported 22 differentially expressed microRNAs and 1561 differentially expressed genes in a low-Cd-accumulating wheat cultivar through small RNA and transcriptome sequencing [71] (Figure 2a). Their study indicated that microRNAs play an important role in wheat under Cd stress via regulation of their target genes. Moreover, through ICP-MS-assisted ionomic analysis, it has been reported that low-Fe stress significantly limited the absorption of nutrients, including N, P, Mg, Ca, K, Fe, Mn, Zn, Cu and B nutrients [72] (Figure 2b). They also identified 378 and 2619 differentially expressed genes in the shoots and roots of wheat under low-Fe conditions, respectively. Their results provide excellent ionomic changes in mineral composition in response to low Fe stress and genetic resources for the modulatory mechanisms underlying adaptability to low Fe stress in wheat. In addition, recently, it has been reported through combining ionomics, transcriptomics and functional gene analysis that an important Cd exporter, *TaHMA2b-7A*, modulates long-distance Cd translocation in wheat [110] (Figure 2c). Although great progress has been made in the study of wheat under heavy metal stress through multi-omics, substantial efforts are still needed to explore key functional genes in wheat in response to heavy metal stress.

## 7. Functional Genes and Heavy Metal Stress in Wheat

**Figure 3:**
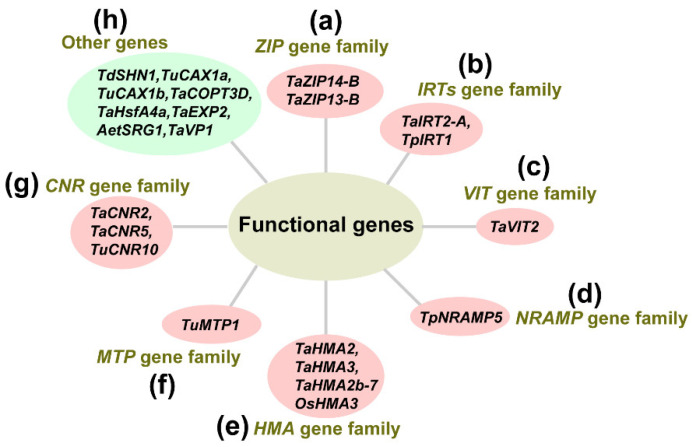
Functional genes that regulate heavy metal stress in wheat.

Initially, through using wheat genome information, researchers commonly identified different gene families regulating heavy metal stress, such as the Zn-regulated, iron-regulated transporter-like protein (ZIP) transporter family [111], zinc-induced facilitator-like (ZIFL-like) gene family [44], vacuolar iron transporters (VIT) family [112], natural resistance-associated macrophage protein (NRAMP) family [113], metal tolerance protein (MTP) family [114] and heavy metal ATPase (HMA) family [71]. Then, through analyzing the phylogenetic trees, chromosomal locations, conserved motifs and expression levels under heavy metal stresses of these gene families, researchers further selected a few potential functional genes to explore their detailed functions under heavy metal stress. With the development of research methods for gene function, the function of certain genes has been well documented (Table 1). Here, we focus on reported functional genes in wheat (not only in common wheat) under heavy metal stress (Figure 3).

The *ZIP* transporter family contributes to regulating the uptake, transport and accumulation of metal trace elements. In the plant, *ZIP* transporters participate in transporting iron and metallic ions. The proteins of *ZIP* transporter genes can transport distinct divalent cations such as Zn^2+^, Fe^2+^, Cd^2+^, Cu^2+^, Mn^2+^, Co^2+^ and Ni^2+^ [111,115]. *TaZIP14-B*, *TaZIP13-B* and *TaIRT2-A* have been reported to transport Zn and Fe through yeast complementation analysis (Figure 3a,b). These results also indicated that overexpression of *TaZIP13-B* revealed better tolerance to Fe/Zn stress in transgenic *Arabidopsis* plants and that they could accumulate more metallic elements in their seeds compared with wild-type *Arabidopsis* [111]. Another study reported that 15 putative *TaZIFL-like* genes were identified by the genome-wide analyses. Under Zn and Fe stress, *TaZIFL2.3*, *TaZIFL4.1*, *TaZIFL4.2*, *TaZIFL5*, *TaZIFL6.1* and *TaZIFL6.2* were increased. Moreover, *TaZIFL4.2* and *TaZIFL7.1* were upregulated under different heavy metals stressors such as Ni, Cd and Co, while *TaZIFL5* and *TaZIFL6.2* remained almost uninfluenced [44]. *Iron-regulated transporters* (*IRTs*) are the primary Fe transporters and members of the *ZIP* family transporters [116]. Overexpression of *TpIRT1* (from Polish wheat (*Triticum polonicum* L.)) in *Arabidopsis thaliana* has been reported to enhance the concentration of Fe, Mn, Co and Cd in tissues and improve plant growth under Fe, Mn and Co deficiencies, while enhancing the sensitivity to Cd more than in the wild type [117] (Figure 3b).

The *VIT* family plays a vital role in maintaining Fe in the suitable physiological range and limiting cellular toxicity [118]. Two *VIT* paralogs, Ta*VIT1* and Ta*VIT2*, exist in the wheat genome which reveal a different expression pattern but are both low in the endosperm. *TaVIT2* (not *TaVIT1*) facilitates the transport of Fe and Mn in yeast. Moreover, overexpression of *TaVIT2* in the endosperm of wheat especially enhanced the Fe concentration in white flour [118] (Figure 3c). Recently, thirty-one *VIT* families of wheat were identified from hexaploidy wheat with the highest number of genes localized on chromosome 2. These results also indicated that most of the *TaVTL* genes were increased in a tissue-specific manner under Mn, Zn and Cu deficiency [112]. While functional experiments about *TaVTL* genes under heavy metal stress need to be further performed; *TaIRT1b-4A*, *TaNAS2-6D*, *TaNAS1a-6A*, *TaNAS1-6B* and *TaNAAT1b-1D* might serve as key regulators in the adaptive responses of wheat under Fe deficiency [72].

The *NRAMP* family serves as metal/H^+^ symporters and can transport divalent metal cations (Zn^2+^, Fe^2+^, Co^2+^, Ni^2+^, Cd^2+^, Cu^2+^ and Mn^2+^) into the cytosol. It has been reported that *TaNRAMP1* was initially identified through genome-wide identification in wheat. The expression of *TaNRAMP1* was significantly reduced in the roots of genome A and B but was constitutively expressed in the genome under an Mn-deficiency [113]. Another study revealed that *TpNRAMP5* from dwarf Polish wheat increased the accumulation of Cd, Mn and Co, but not Fe and Zn [119] (Figure 3d).

Plant *MTPs* mainly transport Zn, Mn and Fe metals, but they also have affinities for other divalent cations such as Cd or Ni [120,121]. *MTPs* are classified according to their putative specificity for transported metal ions, as either of the Mn-, Zn- or Fe/Zn-cation diffusion facilitator family (CDF) type [122]. Twenty *TaMTPs* were identified in wheat, among them, *TaMTP1s* and *TaMTP8s* belonging to Zn-CDFs, *TaMTP2s* to Fe/Zn-CDFs and *TaMTP3-7s* to Mn-CDFs [114]. Another study reported that *TuMTP1* (*Triticum urartu MTP1*) might sequester excess cytosolic Zn^2+^ and Co^2+^ into yeast vacuoles to maintain Zn^2+^ and Co^2+^ homeostasis [123] (Figure 3f).

*HMAs*, known as *P1B-type ATPase*, had an important role in metal transport in plants [124,125,126]. Wheat *TaHMA2* can transport Cd^2+^ and Zn^2+^ across membranes [127] (Figure 3e). Overexpression of *TaHMA2* and the *TaHMA2* derivative (glutamic substituted for alanine from CCxxE) in Arabidopsis enhanced root length, fresh weight and increased Cd^2+^/Zn^2+^ root-to-shoot translocation compared to the wild type [127]. Another study indicated that heterologous expression of *TaHMA3* genes in yeast revealed no transport activities for Cd, which probably explains the low Cd sequestration in wheat roots and then the high Cd translocation to wheat shoots. This was documented by Zhang et al.; they found that overexpression of the *OsHMA3* gene limited root-to-shoot Cd translocation in wheat and Cd accumulation in wheat grain (reduced by nearly 10-fold and by 96%, respectively) [128] (Figure 3e). In addition, another study reported that *TaHMA3* and *TaVP1* encoding proteins related to Cd compartmentalization were significantly increased in roots in under Cd stress, which was associated with enhanced Cd tolerance in wheat and decreased Cd translocation to aboveground parts [37] (Figure 3e,h).

Except for the above gene families, there are also other gene families or genes that play an important role in wheat under heavy metal stress. For instance, the *cell number regulator* (*CNR*) gene family plays an important role in wheat under heavy metal stress. *TaCNR2* is similar to plant cadmium resistance protein, participating in modulating heavy metal translocation. Overexpression of *TaCNR2* in Arabidopsis and rice increased its stress tolerance to Cd, Zn and Mn, and overexpression in rice enhanced Cd, Zn and Mn translocation from roots to shoots [129] (Figure 3g). Another study indicated that overexpression of *TaCNR5* in *Arabidopsis* increased Cd translocation from roots to shoots [130]. In addition, overexpression of *TuCNR10* (isolated from diploid wheat (*Triticum urartu*)) in *Arabidopsis* and rice increased Cd, Mn and Zn tolerance and promoted Cd, Mn and Zn translocation from roots to shoots. Rice overexpressing *TuCNR10* revealed lower Cd and higher Mn and Zn contents in grains than wild-type rice [131] (Figure 3g). Other studies indicated that some genes also participated in wheat under heavy metal stress. For instance, overexpression of durum wheat *TdSHN1* in transgenic yeast and tobacco conferred Cd, Cu and Zn tolerances for phytoremediation of heavy metal-contaminated soils through increasing activities of superoxide dismutase and catalases [132] (Figure 3h). *TuCAX1a* and *TuCAX1b*, two cation/H^+^ antiporters, were isolated from the diploid wheat (*Triticum urartu*). Functional experiments indicated that overexpression of *TuCAX1a* and *TuCAX1b* enhanced Ca^2+^ and Zn^2+^ translocation, increased Ca^2+^, Zn^2+^, Mn^2+^ and Fe^2+^ accumulation when exposed to Cd^2+^ and promoted the tolerance of *Arabidopsis* to exogenous Ca^2+^ and Zn^2+^ [133] (Figure 3h). The wheat copper transporter gene *Cu transporter 3D* (*TaCOPT3D*)-overexpressing lines had more biomass and low root, shoot and grain Cd contents under 20 mM Cd treatment than the wild-type plants. These results indicated that *TaCOPT3D* might be a potential candidate for reducing Cd content in wheat via genetic engineering [134] (Figure 3h). Shim et al. found that *heat shock transcription factor A4a* (*TaHsfA4a*) of wheat and rice conferred Cd tolerance by upregulating metallothionein gene expression in planta [135] (Figure 3h). Another study indicated that overexpression of *TaEXPA2* improved Cd tolerance due to the enhanced activities of H^+^-ATPase, V-ATPase and PPases which helped in conferring Cd tolerance [136] (Figure 3h). Recently, Wei et al. found that overexpression of *AetSRG1* (encoding a Fe(II)/2-oxoglutarate dependent dioxygenase) can decrease Cd accumulation and electrolyte leakage, increase reactive oxygen species production and promote the synthesis of endogenous salicylic acid through interaction with phenylalanine ammonia lyase (PAL) in wheat [137] (Figure 3h). Their results suggest that different genes may be involved in heavy metal detoxification and reactive oxygen species in wheat under Cd stress.

In conclusion, although great progress has been made in exploring functional genes in wheat under heavy metal stress, substantial efforts are still required to find more functional genes that regulate heavy metal stress in wheat. It is also necessary to investigate whether these functional genes are involved in heavy metal reduction (accumulation) in wheat plants, as well as utilize these functional genes to improve the phytoremediation ability of wheat via genetic engineering. Genetic manipulation of these functional genes can be applied to limit the transfer of heavy metals from soil to wheat grains in the future, thereby protecting human health.

**Table 1:** Functional genes in wheat under heavy metal stress.

Gene	Function	References
*TaZIP14-B*	Transport Zn and Fe	[111]
*TaZIP13-B*	Transport Zn and Fe	[111]
*TpIRT1*	Enhance the concentration of Fe, Mn, Co and Cd	[117]
*TaIRT2-A*	Transport Zn and Fe	[111]
*TaVIT2*	Promote the transport of Fe and Mn	[118]
*TpNRAMP5*	Increase the accumulation of Cd, Mn and Co, but not Fe and Z	[119]
*TuMTP1*	Sequester excess cytosolic Zn^2+^ and Co^2+^	[123]
*TaHMA2*	Transport Cd^2+^ and Zn^2+^ across membranes and increase root–shoot Zn/Cd translocation	[54,127]
*TaHMA2b-7A*	Modulate long-distance Cd translocation in wheat	[110]
*OsHMA3*	Limit root-to-shoot Cd translocation and Cd accumulation in wheat	[128]
*TaHMA3* and *TaVP1*	Increase Cd tolerance in wheat and decrease Cd translocation to aboveground parts	[37]
*TaCNR2*	Increase Cd, Zn and Mn tolerance and enhance Cd, Zn and Mn translocation from roots to shoots	[129]
*TaCNR5*	Increase Cd translocation from roots to shoots	[130]
*TuCNR10*	Enhance Cd, Mn, and Zn tolerance	[131]
*TdSHN1*	Confer Cd tolerances by increasing activities of superoxide dismutase and catalases	[132]
*TuCAX1a* and *TuCAX1b*	Increase Ca^2+^, Zn^2+^ translocation	[133]
*TaCOPT3D*	Reduce root, shoot and grain Cd accumulation	[134]
*TaHsfA4a*	Confer Cd tolerance by upregulating metallothionein gene expression	[135]
*TaEXPA2*	Improve Cd tolerance by enhancing activities of H^+^-ATPase, V-ATPase and PPase	[136]
*AetSRG1*	Reduce Cd accumulation	[137]

## 8. Conclusions and Perspectives

Heavy metals have been substantially increasing in the environment to severely influence the growth and quality of wheat. They have been considered as a potential threat to agriculture and human health. Excess heavy metals exhibit harmful effects on wheat plants at cellular and molecular levels through enhanced oxidizing agents, overproduction of lipid peroxidation and production of genotoxicity. Moreover, these heavy metals also actively participate in regulating protein synthesis enzymes (such as proteases and chaperones) to perturb protein metabolism, resulting in suppressed protein biosynthesis. To face these situations, wheat plants evoke either avoidance of uptake or tolerance mechanisms including enhancement of the activities of antioxidant enzymes, regulation of ion homeostasis, activation of certain genes and production of stress proteins. Importantly, the minimization of heavy metal pollution in wheat is urgently needed around the world. At present, several strategies, such as selecting low-heavy-metal-accumulating wheat cultivars, the use of inorganic and organic amendments and exogenous application of PGRs as well as nanoparticles, have been successfully applied to minimize heavy metal toxicity in wheat at the experimental stage.

**Figure 4:**
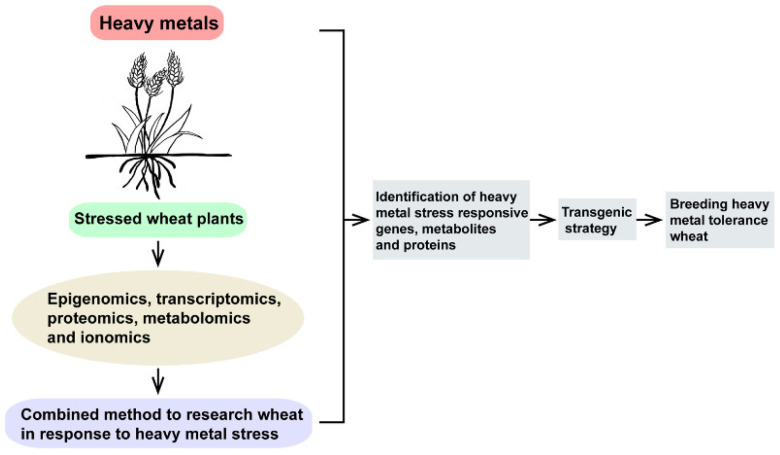
Combined method to investigate wheat plants in response to heavy metal stress. Epigenomics, transcriptomics, proteomics, metabolomics and ionomics are useful methods that can help us to clarify and analyze active regulatory networks regulating heavy metal stress responses and tolerance in wheat plants.

Omics such as epigenomics, transcriptomics, proteomics, metabolomics and ionomics have been applied to depict the mechanisms of wheat–heavy metals interaction at the cellular and molecular levels. The recent study by omics techniques on the effects of heavy metals on wheat development, and how wheat plants respond (tolerate/resist/adapt) to heavy metal stress are summarized. Multi-omics make it possible to comprehensively understand heavy-metal-induced dramatic changes at the genome, transcriptome, proteome, metabolome and ionome levels in wheat. Although some progress has been obtained in epigenetic responses to heavy metal stress in wheat, substantial efforts are still required to investigate other epigenetic responses to heavy metal stress in wheat, such as RNA m^6^A modification and chromatin remodeling. It is also necessary to investigate the epigenetic response to other heavy metal stress, such as Cu, Zn and Fe stress, in wheat, explore epiQTLs in wheat under heavy metal stress, as well as editing the epigenome of wheat by CRISPR-Cas9 for improved heavy metal stress tolerance. Moreover, various studies depicted differentially expressed genes in response to heavy metal stress in wheat. However, the key genes participating in wheat under heavy metal stress are poorly studied. More work is urgently needed to figure out these issues. Furthermore, more work is also needed to figure out the proteomic, metabolomic and ionomic changes in wheat under heavy metal stress, such as Zn, Fe and Cu stress. Substantial efforts are still needed to investigate the connection between cellular mineral nutrients and living systems in wheat under heavy metal stress. In addition, it is also necessary to identify more heavy metal stress-associated functional genes, metabolites and proteins through combining multi-omics analyses, as well as to explore how to apply transgenic strategies to breed heavy metal tolerance in wheat (Figure 4).
